# Blastic Plasmacytoid Dendritic Cell Neoplasm in Long-Term Complete Remission After Venetoclax Monotherapy

**DOI:** 10.7759/cureus.52446

**Published:** 2024-01-17

**Authors:** Yohei Sasaki, So Murai, Eisuke Shiozawa, Toshiko Yamochi, Norimichi Hattori

**Affiliations:** 1 Division of Hematology, Department of Medicine, Showa University School of Medicine, Tokyo, JPN; 2 Department of Pathology, Showa University School of Medicine, Tokyo, JPN

**Keywords:** myc, maintenance therapy, azacitidine, venetoclax, bpdcn

## Abstract

Blastic plasmacytoid dendritic cell neoplasm (BPDCN) is a rare and aggressive hematological malignancy associated with a poor prognosis and limited treatment options. Although allogeneic hematopoietic stem cell transplantation or intensive chemotherapy prolongs overall survival in patients with BPDCN, intensive chemotherapy is inappropriate for older or unfit patients. Venetoclax (VEN), an oral BCL2 inhibitor, is approved for use in patients with acute myeloid leukemia (AML). BPDCN cells require BCL2 protein and are uniformly sensitive to VEN in vivo. Moreover, patients with AML who have achieved complete remission after induction therapy are reportedly considered to receive VEN monotherapy as maintenance therapy, especially older patients. However, the efficacy of VEN monotherapy as a maintenance therapy for patients with BPDCN remains controversial. Recently, BPDCN has been classified into MYC+ and MYC- subtypes, which show clinical differences. Hence, BPDCN treatment strategies based on the MYC classification may be necessary. Here, we report a case of MYC- BPDCN in an older patient in long-term complete remission after VEN monotherapy following VEN and azacitidine induction chemotherapy.

## Introduction

Blastic plasmacytoid dendritic cell neoplasm (BPDCN), derived from immature plasmacytoid dendritic cells, is a rare, clinically aggressive hematological malignancy that occurs in older men and is frequently characterized by cutaneous, lymph node, and bone marrow (BM) manifestations [1]. It has no standard therapy, and patients empirically receive chemotherapy regimens generally used for the treatment of acute myeloid leukemia (AML) or acute lymphoid leukemia (ALL). However, most patients with BPDCN relapse after the initial chemotherapy, and the median overall survival is only 12-14 months [1].

Venetoclax (VEN), an oral BCL2 inhibitor, in combination with hypomethylating agents (HMA) or low-dose cytarabine, is approved for older patients with AML [2,3]. A recent study demonstrated that BPDCN cells target BCL2 proteins and are uniformly sensitive to VEN in vivo [4]. In addition, VEN-combined chemotherapy is reportedly effective in patients with primary and relapsed BPDCN [5,6].

We present the case of a patient with BPDCN who achieved a complete response with an initial therapy of VEN in combination with azacytidine (AZA) and has remained in complete remission (CR) with VEN monotherapy.

## Case presentation

**Figure 1:**
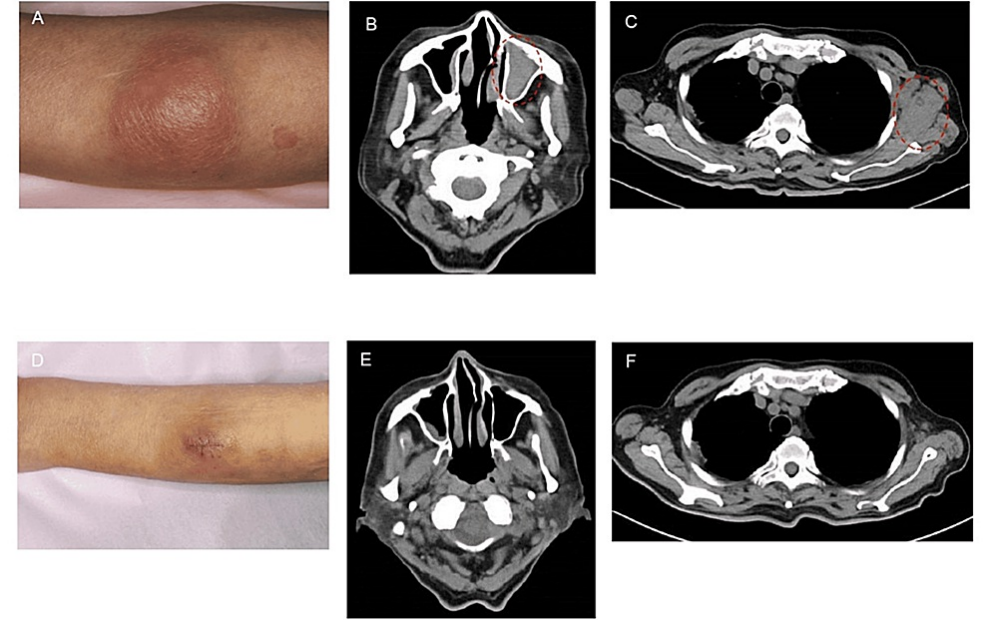
Subcutaneous tissue of the left antebrachium, soft shadow in the left maxillary sinus on cervical computed tomography before and after venetoclax/azacitidine treatment. A subcutaneous tissue of the left antebrachium with a size of 60 × 30 mm (A), a soft shadow in the left maxillary sinus on cervical computed tomography (CT) (B), and a mass in the left axilla on thoracic CT (C) observed on admission. After the first venetoclax/azacitidine therapy cycle, a scar on the subcutaneous tissue with a size of 28 × 24 mm (D) was observed, and soft shadow (E) and mass in the left axilla (F) disappeared.

An 82-year-old man with type 2 diabetes, dyslipidemia, and retinitis pigmentosa (total blindness at 45 years of age), without any subjective symptoms such as fever, night sweat, or weight loss, presented with a skin mass on his left forearm (Figure 1, Panel A).

He had no lymphadenopathy or hepatosplenomegaly. His vital signs were tympanic temperature, 36.8°C (98.24°F); blood pressure, 144/87 mmHg; pulse, 87 beats/minute; respiratory rate, 18 breaths/minute; and oxygen saturation, 98% on room air. His white blood cell (WBC) count was 4.5 × 10^3^/µL, with 55.5% pathological cells. His lactate dehydrogenase and soluble IL-2 receptor (sIL-2R) levels were 393 and 1,087 U/mL, respectively (Table 1).

**Table 1:** Laboratory data on admission. WBC: white blood cells; Seg: segmented neutrophils; RBC: red blood cells; Hb: hemoglobin; Ht: hematocrit; PLT: platelets; TP: total protein; Alb: albumin; T. Bil: total bilirubin; D. Bil: direct bilirubin; BUN: blood urea nitrogen; Cre: creatinine; AST: aspartate aminotransferase; ALT: alanine aminotransferase; LDH: lactate dehydrogenase; ALP: alkaline phosphatase; γ-GTP: gamma-glutamyl transferase; CK: creatine kinase; CRP: C-reactive protein; s-IL2R: soluble interleukin-2 receptor; WT-1: Wilms tumor gene 1; Ig: immunoglobulin; PT: prothrombin time; APTT: activated partial prothrombin time; Fib: fibrinogen

Peripheral blood	Result	Unit
WBC	45,400	/μL
Seg	12	%
Stab cells	0.5	%
Monocytes	6.5	%
Lymphocytes	24	%
Pathological cells	55.5	%
RBC	493 × 10^4^	/μL
Hb	13.1	g/dL
Ht	39	%
PLT	8.2 × 10^4^	/μL
Blood chemistry		
TP	7.5	g/dL
Alb	4.4	g/dL
T. Bil	0.8	mg/dL
D. Bil	<0.1	mg/dL
BUN	14.8	mg/dL
Cre	0.73	mg/dL
AST	27	IU/L
ALT	16	IU/L
LDH	393	IU/L
ALP	59	IU/L
γ-GTP	40	IU/L
CK	23	IU/L
Na	135.1	mEq/L
K	4	mEq/L
Cl	100.4	mEq/L
Ca	9.3	mg/dL
i-P	2.7	mg/dL
CRP	0.66	mg/dL
s-IL2R	1087	U/mL
WT-1	<50	copy/μgRN
IgG	1436	mg/dL
IgA	256	mg/dL
IgM	135	mg/dL
Coagulation		
PT	87	%
APTT	31.5	seconds
Fib	452	mg/dL
D-dimer	0.92	μg/mL

**Figure 2:**
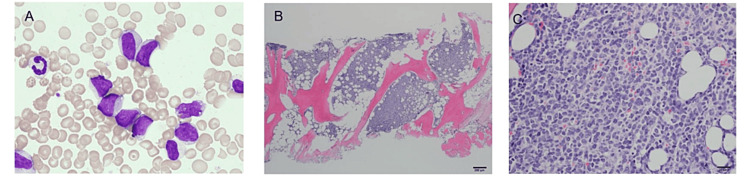
Bone marrow smears before treatment. Bone marrow smears show medium-to-large-sized pathological cells with vacuoles and granules with round or irregular nuclei and a nuclear/cytoplasm ratio of 60–80% (A, ×400). Histopathological examination of the bone marrow on hematoxylin and eosin-stained images shows diffuse infiltration of the pathological cells (B, ×40; C, ×400).

Computed tomography (CT) revealed a soft shadow in the left maxillary sinus and multiple masses extending from the left axilla to the supraclavicular fossa (Figure 1, Panels B, C). The smear of BM aspirates revealed a nucleated cell count of 13.2 × 10^4^/µL, with 74.8% pathological cells (Table 2, Figure 2).

**Table 2:** Bone marrow examination findings. NCC: neural crest-derived cells; MgK: megakaryocytes; FCM: flow cytometry; MYC: myelocytomatosis oncogene

Parameter	Result	Unit
NCC	132,000	/μL
MgK	7	/μL
Erythroid	4.8	%
Myeloid	11.8	%
Promyelocytes	0.6	%
Myelocytes	0.8	%
Metamyelocytes	0.9	%
Stab cells	2	%
Segmented cells	7.2	%
Monocytes	2.3	%
Lymphocytes	6.2	%
Pathological cells	74.8	%
FCM (69.7% gating)		
CD4	88.9	%
CD56	90.4	%
CD123	97.0	%
TdT	67.5	%
MYC split signal	(-)	
G-band	46, XY	

**Figure 3:**
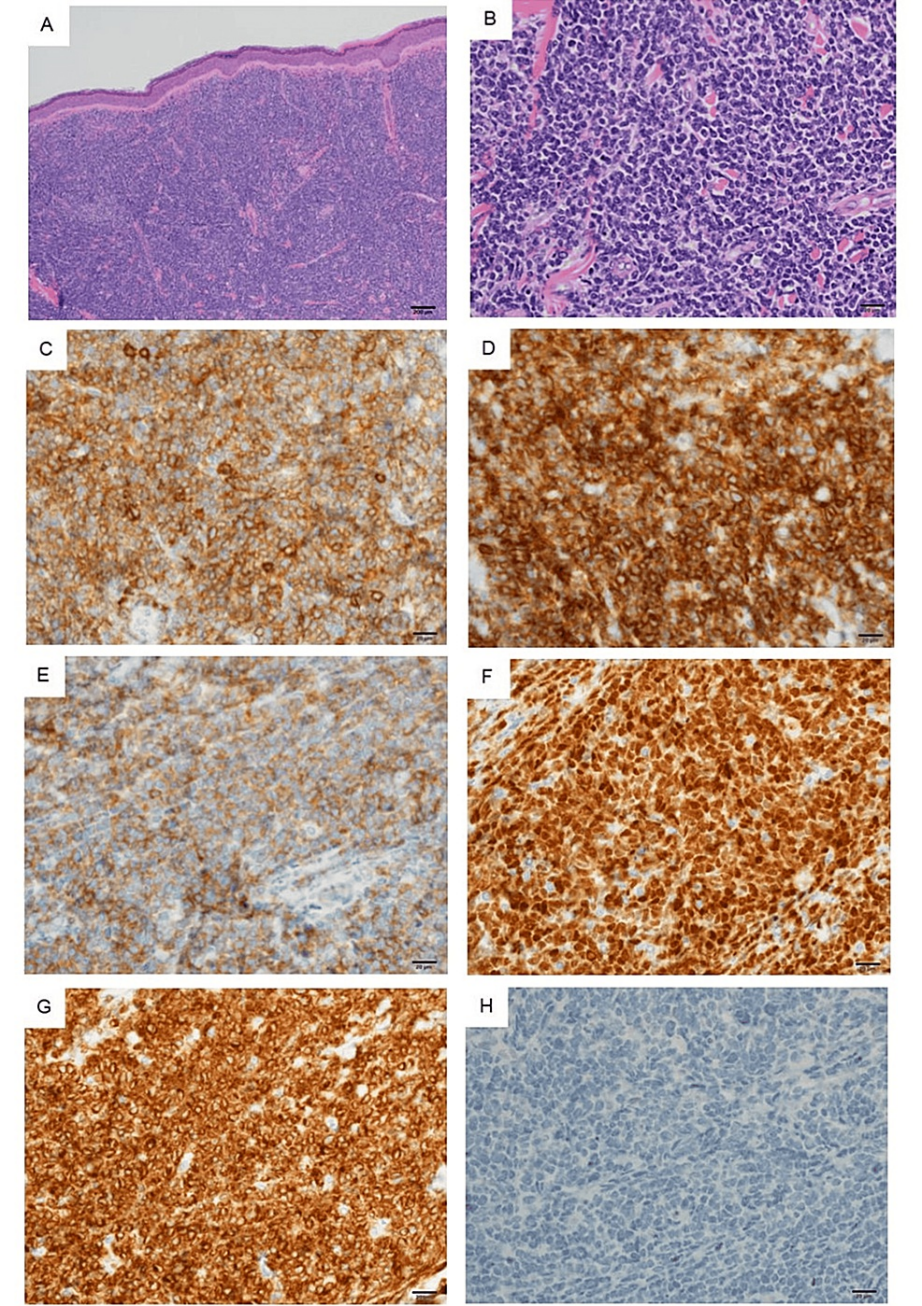
Histopathological examination of pathological cells. Histopathological examination of pathological cells stained with hematoxylin and eosin shows diffuse infiltration (A, ×40; B, ×400). Immunohistochemical staining shows pathological cells with high CD4 (C, ×400), CD56 (D, ×400), CD123 (E, ×400), TdT (F, ×400), and BCL2 (G, ×400) and low MYC (H, ×400) protein expression.

Flow cytometry and immunohistochemistry of the BM and skin biopsies revealed the following immunophenotypes: cluster of differentiation 4 (CD4)(+), CD56(+), CD123(+), terminal deoxynucleotidyl transferase (TdT)(+), BCL2(+), CD3(-), CD5(-), CD20(-), CD79a(-), MPO(-), and MYC(-). MYC rearrangements were not detected (Figure 3).

Karyotype analysis revealed a normal male pattern in the pathological cells. Ultimately, the patient was diagnosed with BPDCN.

**Figure 4:**
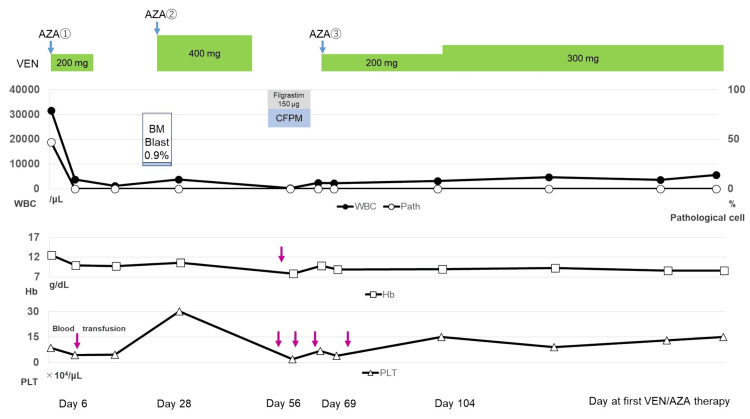
Course of treatment and blood count of the patient. Six days after the initiation of VEN/AZA therapy, pathological cells were not detected in the peripheral blood. Twenty-eight days after the initiation of VEN/AZA therapy, the therapeutic efficacy was evaluated as a complete response based on bone marrow testing and computed tomography findings. Fifty-six days after the initiation of VEN/AZA therapy, the patient developed febrile neutropenia and received a subcutaneous injection of filgrastim and antimicrobial therapy with cefepime. Sixty-nine days after the initiation of VEN/AZA therapy, while the patient received the third cycle of VEN/AZA therapy, subcutaneous AZA injections were discontinued due to bone marrow suppression. The patient was started on VEN monotherapy at a dose of 200 mg/day, which was increased to 300 mg/day mg/day 104 days after the initiation of VEN/AZA therapy. The patient continued to receive VEN monotherapy for 15 months without any adverse events or relapse. AZA: azacitidine; VEN: venetoclax; CFPM: cefepime; BM: bone marrow; WBC: white blood cells; PLT: platelets; Hb: hemoglobin

He was admitted to our hospital in August 2022. Although he had comorbidities, including diabetes, dyslipidemia, and retinitis pigmentosa, his Eastern Cooperative Oncology Group performance status score was 1. However, he was not deemed suitable to undergo intensive chemotherapy due to his age. Therefore, we selected treatment with VEN and AZA. The patient provided informed consent for treatment comprising VEN and AZA, a drug approved for AML. The treatment comprised VEN/AZA (75 mg/m^2^) on days one to seven. The VEN dose was 100 mg/day on day one and was adjusted to 200 mg/day on day two, considering the risk of tumor lysis syndrome. Six days after the start of VEN/AZA therapy, the pathological cells disappeared from the peripheral blood. Sixteen days after the start of VEN/AZA therapy, the patient discontinued VEN due to the development of neutropenia and thrombocytopenia. Twenty-eight days after the start of VEN/AZA therapy, the patient had recovered from BM suppression. BM examination results showed the disappearance of pathological cells, while CT showed the disappearance of a soft shadow in the left maxillary sinus and multiple masses (Figure 1, Panels D-F). Although the patient did not undergo positron emission tomography-CT examination, CR was determined based on the results of the BM examination, CT findings, and decreased sIL-2R level (594 U/mL). The patient then received a second cycle of VEN/AZA therapy, during which the VEN dose was increased to 400 mg/day. However, VEN was discontinued 20 days after the second cycle of VEN/AZA due to the development of BM suppression and febrile neutropenia (FN). After recovering from BM suppression and FN, the patient was administered a third cycle of VEN/AZA therapy with a reduced dose of AZA. Nevertheless, the patient developed BM suppression, which required treatment. The repeated BM suppression suggested that the patient was unable to tolerate the VEN/AZA therapy. Given the BPDCN cell sensitivity to VEN, we discontinued AZA on day three of the third cycle of treatment and continued VEN at a dose of 200 mg/day. No significant adverse events were observed, and the VEN monotherapy dose was increased to 300 mg/day 35 days after the third cycle of VEN/AZA therapy. The patient showed a complete response to BPDCN and was prescribed VEN monotherapy as a maintenance therapy for CR. He has continued VEN monotherapy and has remained in CR with normal s-IL2R values for 15 months since the BPDCN diagnosis (Figure 4).

## Discussion

Although allogeneic hematopoietic stem cell transplantation (AHSCT) or intensive chemotherapy for ALL, such as using hyper-central venous access devices, prolongs the overall survival of patients with BPDCN [7,8], these therapies are inappropriate for older patients or for those who are physically unfit; thus, the prognosis of older or physically unfit patients with BPDCN is poor.

VEN/AZA therapy is considered beneficial for the treatment of older patients with AML (aged >75 years) [3]. Montero et al. reported that BPDCN cells are more dependent on BCL2 and are more sensitive to VEN than AML cells [4]. Several studies have demonstrated the effectiveness of AZA therapy in patients with BPDCN [9,10]. Therefore, VEN/AZA therapy may be an appropriate treatment option for patients with BPDCN. Gangat et al. reported on five patients with primary BPDCN and five patients with relapsed BPDCN who received VEN-combined HMA therapy, in which VEN-combined HMA therapy led to CR in patients with BPDCN [5]. In addition, a previous case report described a patient with BPDCN who received continuous VEN/AZA therapy and remained in CR [6]. However, because patients treated with VEN/AZA therapy often present with FN, several patients, especially older adults or those who are unfit, discontinue VEN/AZA therapy [11,12]. A previous study considered VEN as maintenance therapy for patients with AML who achieved CR after induction therapy with VEN/AZA, especially older patients [13]. Our patient presented with FN during the second cycle of VEN/AZA therapy and repeated BM suppression. Therefore, he continued VEN monotherapy instead of VEN/AZA therapy after achieving CR. The median overall survival for patients with BPDCN is only 12-14 months despite intensive chemotherapy. Our patient has remained in CR for 15 months. A previous study showed that the median overall survival of patients with BPDCN, aged over 65 years, was 7.1 months [7]. The continued VEN monotherapy may explain why our patient has remained in CR. However, reports on VEN monotherapy for BPDCN are limited. Previous studies have reported two cases of VEN monotherapy as salvage therapy for relapsed BPDCN [4]. In both cases, the therapeutic efficacy of VEN monotherapy for relapsed BPDCN was limited. However, to our knowledge, VEN monotherapy as a maintenance therapy for BPDCN has not been reported. Further studies including diverse patient populations are needed to establish this maintenance therapy for older or physically unfit patients with BPDCN.

Sakamoto et al. proposed the classification of BPDCN based on the MYC gene and protein into immunoblastoid BPDCN (MYC+ BPDCN) and classical BPDCN (MYC- BPDCN) subtypes and described the different clinical characteristics of MYC+ BPDCN and MYC- BPDCN [14]. Thus, BPDCN may have different treatment strategies according to these subtypes. Moreover, the authors reported poorer outcomes in patients with MYC+ BPDCN than those with MYC- BPDCN. The poorer outcomes in patients with MYC+ BPDCN might be due to the lower age and higher proportion of patients who underwent AHSCT among those with MYC- BPDCN compared with those with MYC+ BPDCN. This finding suggests that AHSCT is an effective treatment for younger patients with MYC- BPDCN. However, treatment strategies for MYC- BPDCN in older or physically unfit patients remain unclear. Thus, we suggest that, in patients with MYC- BPDCN who achieve CR following initial therapy, AHSCT might be recommended as consolidation therapy for young and physically fit patients, while VEN monotherapy might be recommended as maintenance therapy for older or physically unfit patients. Further research is required to validate this proposed strategy. In addition, studies are also needed to verify our proposed strategy in patients with MYC+ BPDCN.

## Conclusions

BPDCN is a rare disease with a poor prognosis; thus, an appropriate treatment strategy is required to improve patient prognosis. However, there is still no consensus on BPDCN treatment in older or physically unfit patients with BPDCN. Our case report demonstrated that VEN/AZA induction therapy and VEN monotherapy as maintenance therapy after complete response are potentially appropriate strategies for older or physically unfit patients with MYC- BPDCN.
